# Optical Design of a Large-Angle Spectral Confocal Sensor for Liquid Surface Tension Measurement

**DOI:** 10.3390/s26020599

**Published:** 2026-01-15

**Authors:** Lingling Wu, Tingting Yang, Fang Wang, Qian Wang, Fei Xi, Jinsong Lv

**Affiliations:** 1 School of Optoelectronic Engineering, Xi’an Technological University, Xi’an 710021, China; yangtingting284@163.com (T.Y.); wangfang86@xatu.edu.cn (F.W.); qianwang@xatu.edu.cn (Q.W.); 2 Xi’an Guide Technology Co., Ltd., Xi’an 710065, China; brandenxi@163.com; 3 Huazhong Institute of Electro-Optics-Wuhan National Laboratory for Optoelectronics, Wuhan 430223, China; lvjinsong1@cssc717.com

**Keywords:** spectral confocal, dispersive objective lens, Czerny–Turner (C-T) spectrometer, surface tension, large-angle measurement

## Abstract

**Highlights:**

**What are the main findings?**

- This study designed a large-angle spectral confocal sensor optical system with 1.5 mm axial dispersion across the 430–700 nm wavelength range and a ±40° wide-angle measurement field, providing optical support for high-precision acquisition of surfaces with large inclination angles.
- A front-end/back-end optical structure was constructed, comprising a dispersion objective lens and a Czerny–Turner (C-T) spectrometer meeting the astigmatism-free condition. The system design and optimization were completed, with its engineering feasibility verified through tolerance analysis.

**What are the implications of the main findings?**

- The proposed spectral confocal sensor optical system has been developed to enable accurate extraction of droplet surface profiles under high-inclination conditions and, combined with the Young–Laplace equation, enables high-precision determination of droplet surface tension.
- The system retains high measurement accuracy over a wide range of angles, providing robust technical support for the precise characterization of high-inclination surfaces and offering a reference framework for the systematic design of large-angle spectral confocal sensors.

**Abstract:**

The surface tension of a liquid droplet can be determined by fitting its actual profiles using the Young–Laplace equation, effectively reducing the measurement of surface tension to an accurate determination of the droplet’s profiles. Spectral confocal sensors are high-precision, interference-resistant, non-contact measurement systems for droplet surface profiling, employing a light source together with a dispersive objective lens and a spectrometer to acquire depth-dependent spectral information. The accuracy and stability of surface tension measurements can be effectively enhanced by spectral confocal sensors measuring the droplet surface profile. Although existing spectral confocal sensors have significantly improved measurement range and accuracy, their angular measurement performance remains limited, and deviations may arise at droplet edges with large inclinations or pronounced surface profile variations. This study presents the optical design of a large-angle spectral confocal sensor. By theoretically analyzing the conditions for generating linear axial dispersion in the dispersive objective lens, a front-end dispersive objective lens was designed by combining positive and negative lenses. Based on a Czerny–Turner (C-T) configuration, the back-end spectrometer was designed under the astigmatism-free condition, taking into account both central and edge wavelength effects. Zemax was employed for simulation optimization and tolerance analysis of each optical module. The results show that the designed system achieves an axial dispersion of 1.5 mm over the 430–700 nm wavelength range, with a maximum allowable object angle of ±40° and a theoretical resolution of 3 μm. The proposed spectral confocal sensor maintains high measurement accuracy over a wide angular range, facilitating precise measurement of droplet surface tension at large inclination angles.

## 1. Introduction

Surface tension normally refers to the interfacial tension at the gas–liquid interface and is intimately related to liquid behaviors such as adsorption, adhesion, wetting, and lubrication. It directly affects the performance of products such as surfactants, emulsifiers, and lubricants [1,2,3]. Furthermore, surface tension has numerous applications in domains such as biomedicine [4,5,6], petrochemical engineering [7,8], and engineering technology [9,10]. In medical diagnostics [11], surface tension can be used to monitor conditions such as enamel caries, blood coagulation, and dry eye syndrome. In petroleum production [12], oil recovery can be improved by adjusting reservoir wettability and surface tension. In engineering applications [13], the surface tension of liquids can enhance the thermal conductivity and heat transfer performance of thermal systems. Moreover, with the trend toward device miniaturization, surface-tension-driven nanofluid flow has shown great potential for microelectronic cooling [14]. Therefore, in-depth investigation of droplet surface tension is of significant theoretical importance and provides essential technical support for practical applications in related fields.

Traditional contact-based surface tension measurement methods, such as the maximum bubble pressure, pendant drop, and Wilhelmy plate methods, are susceptible to contamination and may modify the intrinsic surface properties of the liquid. Optical non-contact measurement techniques have therefore been widely adopted in research due to their minimal perturbation of the measured surface and rapid, real-time detection capabilities. Kerscher [15] employed the static light scattering (SLS) method to measure the viscosity and surface tension of benzene under saturated conditions. By analyzing capillary waves at the liquid–gas interface, the method enables precise droplet measurements without requiring calibration, and the results show good agreement with reference values. However, this method is highly sensitive to air turbulence and vibration. Wu [16] proposed the rainbow refractometry method, in which oscillation parameters are extracted from time-resolved rainbow angular displacements to measure the liquid surface tension. This method allows measurements of evaporating droplets under high-temperature and high-pressure conditions, but it is sensitive to the droplet’s refractive index and surface morphology. Liu [17] introduced a stereoscopic imaging technique for precise measurement of airborne droplets, which does not rely on symmetry assumptions. Nevertheless, edge and defocus blurring lead to reduced measurement accuracy.

With advances in theoretical research and computer-aided techniques, Rotenberg proposed the axisymmetric drop shape analysis (ADSA) method [18], which is based on the Young–Laplace equation and relates the local surface curvature of a droplet to the interfacial pressure difference and surface tension. The ADSA method enables high-precision inversion of droplet surface tension; however, the inversion results are highly sensitive to the accuracy of droplet profile acquisition. Errors in the extracted profile directly affect curvature calculation and consequently degrade the accuracy of surface tension determination. Meanwhile, the droplet surface itself typically contains regions with large local slopes, where measurement errors are more likely to occur, further reducing the accuracy of surface tension inversion. Therefore, accurately obtaining the droplet surface profile in a non-contact manner has become a key issue in surface tension measurement methods based on the Young–Laplace equation.

The spectral confocal method is a non-contact optical profilometry technique that integrates spectral characteristics with confocal principles. Compared with interferometric or imaging-based methods, it enables non-contact, point-wise axial position measurement and exhibits high robustness to variations in surface reflectivity, making it suitable for measurement scenarios that require high accuracy in axial profile acquisition. It provides high spatial resolution, excellent measurement precision, and robust adaptability. Based on this principle, a spectral confocal sensor can be constructed, whose core components are a dispersive objective lens and a spectrometer. As a core element of spectral confocal sensors, the dispersive objective lens has been the subject of extensive investigation. He [19] designed a compact six-element dispersive objective lens providing a 12 mm dispersion range over wavelengths from 450 nm to 656 nm. The objective lens has an object-side numerical aperture (NA) of 0.22 and an image-side NA varying between 0.198 and 0.24. The image-side numerical aperture (NA) characterizes the maximum allowable object angle range of the returned light that can be effectively collected by the dispersive objective lens and therefore constrains the measurable surface inclination of the system. The assembled system achieves a spatial resolution of 0.15 μm. However, its limited measurement angle restricts observations of highly inclined objects. Wang [20] proposed a dispersive objective lens based on a diffractive–refractive hybrid design, featuring an NA range of 0.57–0.615. However, the system is restricted to a measurement range of 514.8 μm, and the integration of diffractive optical elements increases its cost. Bai [21] designed a seven-element dispersive objective lens, achieving an axial dispersion range of 398 µm with an image-side NA of 0.45. However, its overall dispersion capability remains relatively limited. It can be seen that, although researchers have adopted different design approaches for dispersive objective lenses and have made corresponding trade-offs between performance metrics and system complexity, increasing the numerical aperture remains one of the key development directions in dispersive objective lens design.

In the field of spectrometer design, miniaturization and high performance have emerged as the dominant development trends. To address these requirements, researchers have extensively investigated integrated spectrometer architectures and optical performance optimization strategies. Li [22] proposed an inversely designed integrated spectrometer, which achieves a spectral resolution of approximately 0.3 nm while maintaining a compact footprint and low power consumption. Feng [23] designed an ultra-thin Czerny–Turner spectrograph using cylindrical optical elements and a planar grating, achieving a spectral resolution of 0.4 nm at the central wavelength and 0.75 nm at the spectral edges. Guo [24] proposed a modified optical design for a broadband, high-resolution, astigmatism-free Czerny–Turner spectrometer, in which astigmatism is corrected over a broad spectral range by employing cylindrical mirrors. The results show that the RMS spot radius is reduced to 4.2 μm at the central wavelength and 17 μm at the spectral edge wavelengths. In addition, some studies have optimized spectrometer systems from an architectural perspective by improving optical path layouts and detection mechanisms [25,26,27], thereby enhancing measurement accuracy and providing new insights for the performance optimization of compact spectroscopic systems. A compact, high-performance spectrometer is a key component for axial position demodulation in a spectral confocal sensor. Given that spectral resolution has a significant impact on the accuracy of axial position demodulation, this study enhances the spectral resolving capability of the spectrometer through optical path design optimization, thereby supporting improved axial localization accuracy in the spectral confocal sensor.

To meet the requirements for high precision and large-angle capability in droplet surface profile measurements, we propose the design of a large-angle spectral confocal sensor optical system based on spectral confocal microscopy for measuring the surface tension of hydrophilic droplets, thereby achieving accurate characterization of droplet profiles. First, we describe the operating principle of the spectral confocal sensor, followed by a theoretical analysis covering both the dispersive objective lens and the spectrometer under astigmatism-free conditions. Second, we design and optimize the large-angle spectral confocal sensor’s optical system through module-level simulations in Zemax OpticStudio version 2024 R1. Finally, we conduct a tolerance analysis on the dispersive objective lens together with the spectrometer under astigmatism-free conditions. The analysis shows that the system developed in this study can perform large-angle measurements while maintaining high accuracy, demonstrating good feasibility for practical manufacturing applications.

## 2. Working Principle and Parameter Analysis of the System

### 2.1. Working Principle of Static Droplet Surface Tension Measurement

**Figure 1:**
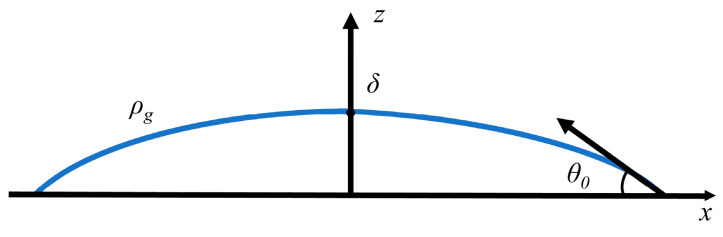
Cross-sectional profile of a static, axisymmetric droplet. Blue line represents the droplet’s cross-sectional profile.

The investigation of droplet profiles forms an essential basis for determining surface tension. To establish a computational model for droplet surface tension, the droplet is assumed to exhibit rotational symmetry under static equilibrium conditions. The corresponding cross-sectional profile is illustrated in Figure 1.

The droplet profile curve can be described using the two-dimensional Young–Laplace equation [28]:

(1) $$\kappa \sigma_{\mathrm{lg}}=\Delta p$$

where κ denotes the curvature of the liquid surface, Δp represents the pressure difference across the liquid–gas interface, and $\sigma_{\mathrm{lg}}$ is the surface tension at the liquid–gas interface.

The pressure difference across the liquid surface can be expressed by the following equation:

(2) $$\Delta p=\rho g[\delta -z]-\rho_{g}$$

In Equation (2), $\rho_{g}$, ρ, g and δ represent atmospheric pressure, droplet density, gravitational acceleration, and droplet height, respectively.

Due to the rotational symmetry of the droplet, the curvature of the liquid surface can be considered as the curvature of the profile curve, which can be expressed by the following equation:

(3) $$\kappa (x)=-{\frac{d^{2}z}{dx^{2}}/\left[1+{\left(\frac{dz}{dx}\right)}^{2}\right]}^{\frac{3}{2}}$$

By solving the differential equation of the droplet profile along with the boundary conditions, an explicit expression for the droplet profile on a horizontal plane can be obtained. Since surface tension is one of the parameters in the explicit profile function, it can be determined by fitting the profile function to the set of droplet profile coordinates [*x*, *z*] on the horizontal plane.

(4) $$\begin{array}{c} x(z)=-\sqrt{\frac{B^{2}-4AC}{4A^{2}}}\mathrm{EllipticE}\left[\sqrt{Az^{2}+Bz+C},\sqrt{\frac{4A}{4AC-B^{2}}}\right]+\left(\frac{B^{2}+2A-4AC}{2A\sqrt{B^{2}-4AC}}\right)\\ \mathrm{EllipticF}\left[\sqrt{Az^{2}+Bz+C},\sqrt{\frac{4A^{2}}{4AC-B^{2}}}\right] \end{array}$$

where $A=\frac{\rho g}{4\sigma_{\mathrm{lg}}}$, $B=-\frac{1}{2}\left(\frac{1}{\delta }+\frac{\rho g\delta }{2\sigma_{\mathrm{lg}}}-\frac{\cos\theta_{0}}{\delta }\right)$, $C=\frac{1-\cos\theta_{0}}{2}$, By fitting the measured droplet surface profile to Equation (4), the values of the coefficients A, B, and C can be obtained. These values can then be used, along with the relevant parameters, to determine the surface tension of the static droplet.

The edges of droplets typically correspond to regions with high local slopes, which are highly sensitive to curvature estimation. Conventional sensors with limited allowable object angle range often fail to fully capture these edge details, introducing significant errors in curvature calculation and consequently increasing the uncertainty in surface tension determination. The article proposes a large-angle spectral confocal optical system to expand the allowable object angle range, thereby enabling more accurate acquisition of droplet profiles—including edge and high-slope regions—reducing profile fitting errors and improving curvature estimation accuracy. Since surface tension is directly related to curvature, errors in profile measurements propagate through the Young–Laplace equation, leading to uncertainties in surface tension calculation. Therefore, quantifying the reduction in curvature errors can directly decrease the uncertainty in surface tension determination. Furthermore, appropriate post-processing algorithms can be applied to further mitigate residual measurement noise, enhancing the reliability and accuracy of surface tension estimation.

### 2.2. Working Principle of the Spectral Confocal Sensors

**Figure 2:**
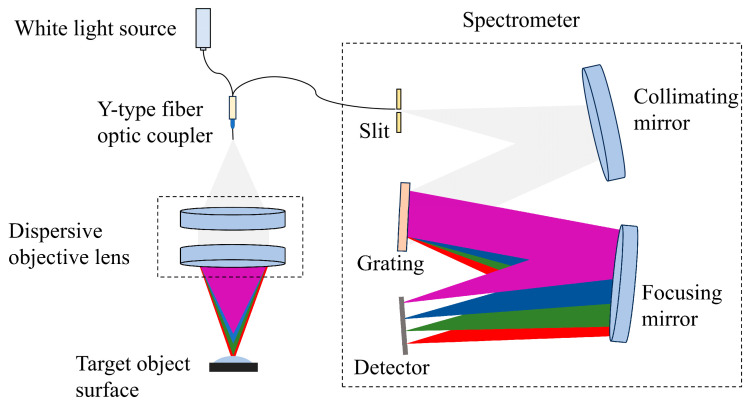
Schematic diagram of the spectral confocal sensor’s working principle. Rays of different colors represent light at different wavelengths, with red corresponding to the longest wavelength and violet to the shortest.

Figure 2 provides an overview of the spectral confocal sensor’s working principle. The core components of the system include a light source, a dispersive objective lens, and a spectrometer. Light emitted from the white light source passes through a Y-type fiber optic coupler and emerges as a point source. As it propagates through the dispersive objective lens, the point source generates a series of focal points along the optical axis, each corresponding to a different wavelength due to chromatic dispersion. When the object lies within the measurement region, only light of a specific wavelength is sharply focused onto the object’s surface. The reflected light then travels back along the same optical path through the dispersive objective lens and reaches the spectrometer’s entrance slit, where it passes with maximum energy. In contrast, light at other wavelengths is defocused on the measured surface and subsequently restricted by the slit after reflection, resulting in reduced energy entering the spectrometer. The spectrometer records a spectral signal exhibiting a pronounced intensity peak at the target wavelength. By decoding the spectral data, a mapping between wavelength and displacement can be established, thereby providing displacement information of the measured surface.

The large-angle spectral confocal sensor utilizes a Thorlabs MWWHF1 white-light LED (light-emitting diode), which provides light across a nominal spectral span from 400 nm to 800 nm. Due to the relatively lower spectral intensity at the edges of the spectrum, the effective working wavelength range is set to 430–700 nm. A Y-type fiber optic coupler directs light from the LED toward the dispersive objective and collects the light returning from the surface of the object under measurement. This arrangement enhances the modular independence of the light source, dispersive objective lens, and spectrometer, and allows for separated optical paths. The measurement range of the system is primarily determined by the axial dispersion introduced by the dispersive objective lens. While increasing the dispersion range can extend the measurable depth, it may also reduce the available light intensity and degrade the signal-to-noise ratio. The ability to perform large-angle measurements is controlled by the image-side NA of the dispersive objective lens. A larger NA increases the maximum allowable object angle and enhances light energy utilization efficiency, while also complicating aberration correction and system configuration, potentially affecting linearity and the overall dispersion range. Based on these trade-offs, the system parameters were optimized, and the final design specifications are summarized in Table 1.

Based on the system configuration and parameter selection, the measurement constraints for droplets are as follows. In the designed optical system, the spectral confocal sensor achieves an axial dispersion range of 1.5 mm, which limits the droplet height to within 1.5 mm. Height variations beyond this range may lead to spectral peak overlap or ambiguity in axial position measurement. The lateral dimension of the droplet primarily depends on the scanning strategy or acquisition method of the imaging system. This study does not impose any specific restriction on the lateral dimension of the droplet.

To ensure stable spectral peak extraction and accurate axial localization, the influence of the local surface slope of droplets on the performance of the confocal optical system must be considered. Previous studies have shown that measurement uncertainty increases with increasing surface slope. Duan [29] reported that in the dispersive confocal probe system they used, when the surface slope approached ±45°, the measurement accuracy deteriorated significantly. This performance degradation was attributed to reduced reflected signal from high-slope regions, resulting in decreased probe signal strength. Considering the practical measurement limitations of the system, this study adopts a conservative upper limit of ±40° as the design reference, which is defined as the maximum allowable object angle of the proposed spectral confocal system. This value aligns with the surface slope characteristics of hydrophilic droplets on glass substrates and helps ensure stable and reliable signal acquisition under practical measurement conditions [30]. In addition, to avoid spectral peak splitting or broadening, the droplet surface should be smooth and possess sufficient optical reflectivity.

In summary, the measurable droplets in this system are expected to satisfy an axial height not exceeding 1.5 mm, a local surface slope whose reflected rays remain within the maximum allowable object angle of approximately ±40°, and a smooth surface with sufficient optical reflectivity. These conditions ensure stable spectral peak extraction and reliable axial positioning under the present system configuration.

### 2.3. Analysis of System Parameters

#### 2.3.1. Analysis of Dispersive Objective Lens Parameters

The dispersive objective lens controls the displacement associated with each wavelength, which defines the sensor’s measurement range. The primary considerations in designing a dispersive objective lens are the axial dispersion range, dispersion linearity, and aberration correction. For a lens group composed of *N* closely spaced thin lenses to achieve dispersion linearity, its dispersion characteristics and optical powers must satisfy the following conditions [31]:

(5) $$\varphi =\sum_{i=1}^{N}\varphi_{i}$$

(6) $$\sum_{i=1}^{n}\frac{\varphi_{di}}{\nu_{di}}=\frac{-\delta s_{CF}^{\prime }}{{f^{\prime }}^{2}{\left(1-a\right)}^{2}}$$

(7) $$\sum_{i=1}^{N}\frac{\varphi_{di}}{\nu_{di}}P_{\lambda i}=0$$

where φ represents the optical power possessed by the dispersive objective lens; $\varphi_{i}$ denotes the optical power associated with the *i*-th lens; *N* represents the overall count of lenses; $\varphi_{di}$ and $\nu_{di}$ represent, respectively, the optical power and the Abbe number of the *i*-th lens at wavelength d; $\delta s_{CF}^{\prime }$ represents the axial chromatic aberration associated with the dispersive objective lens between wavelengths C and F; $f^{\prime }$ and a are the focal length and lateral magnification corresponding to the dispersive objective, respectively; $P_{\lambda i}$ indicates the dispersion characteristic of the glass material, and $P_{\lambda i}$ can be expressed as:

(8) $$P_{\lambda i}=Q_{\lambda i}\frac{\lambda -\lambda_{C}}{\lambda_{F}-\lambda_{C}}$$

where $Q_{\lambda i}=\frac{n_{\lambda i}-n_{iC}}{n_{iF}-n_{iC}}$ corresponds to the relative dispersion exhibited by the material of the *i*-th lens.

According to Equation (7), to achieve dispersion linearity, the dispersive objective lens must be constructed by combining glasses of different types. Dispersive objective lenses generally exhibit negative dispersion ($\delta s_{CF}^{\prime }$ < 0). According to Equation (6), to achieve a larger dispersion range, materials for lenses with positive focal lengths should have as low an Abbe number as possible, whereas materials for lenses with negative focal lengths should have as high an Abbe number as possible. Meanwhile, increasing the focal length can help expand the dispersion range, However, it also enlarges the size of the dispersive objective lens, necessitating a trade-off between dispersion performance and system miniaturization.

Based on the above analysis and considering both the requirements for linear dispersion and the system’s angular specifications, a multi-glass combination strategy is adopted. By appropriately allocating the optical powers among the lenses, the design satisfies the conditions specified in Equations (6) and (7). Concurrently, the core diameter and NA for the Y-type fiber optic coupler are taken into account. The resulting design parameters of the dispersive objective lens are presented in Table 2.

#### 2.3.2. Analysis of Spectrometer Parameters

The spectrometer is a key back-end component of the chromatic confocal system, responsible for receiving the encoded spectral signal and extracting the peak wavelength. Its key performance indicator is the spectral resolution. During the optical design of a C-T type spectrometer, astigmatism has a significant impact on the achievable resolution and must therefore be effectively controlled.

1. Resolution

The spectrometer can image the same target across a continuous spectral range, directly presenting the spectral characteristics of the measured surface. The resolution of a spectrometer is typically specified based on the design specifications of the overall system. Given the axial resolution δL of the spectral confocal sensor system, together with the working wavelength range Δλ, and the system measurement range ΔL, the resolution of the spectrometer can be expressed as follows:

(9) $$\delta \lambda =\frac{\Delta \lambda \times \delta L}{\Delta L}$$

2. Zeroth-Order and First-Order Astigmatism-Free Conditions

**Figure 3:**
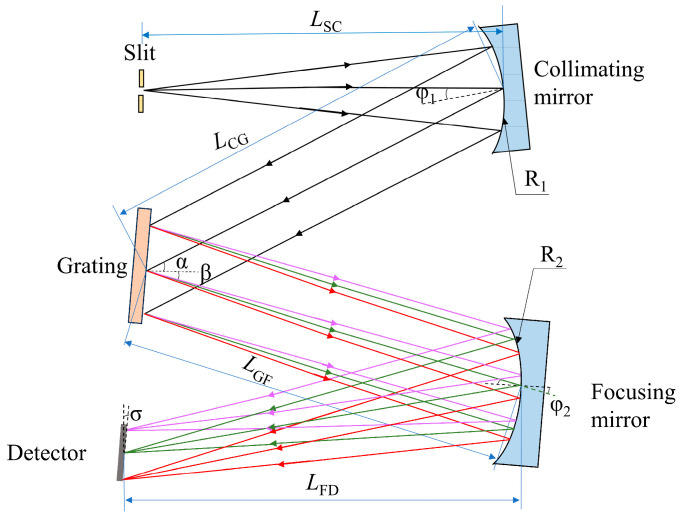
Schematic of the optical path of the C-T type spectrometer. Different colored arrows represent light of different wavelengths, with red corresponding to the longest wavelength and violet to the shortest.

The optical path of the C-T type spectrometer is illustrated in Figure 3. Light emitted from the source passes through an entrance slit into the optical system, is rendered parallel by the collimating mirror, dispersed at the grating, and subsequently brought to focus by the focusing mirror, finally forming an image on the line-scan detector. In the figure, $L_{\mathrm{SC}}$ represents the distance between the entrance slit and the collimating mirror; $\varphi_{1}$ denotes the incident angle at the collimating mirror; $\varphi_{2}$ is the incidence angle of the focusing mirror; $L_{\mathrm{CG}}$ denotes the distance between the collimating mirror and the grating; α and β represent the angles of incidence and diffraction at the grating, respectively; $L_{\mathrm{GF}}$ denotes the distance between the grating and the focusing mirror; $L_{\mathrm{FD}}$ denotes the distance between the focusing mirror and the detector; $R_{1}$ and $R_{2}$ indicate the radii of the collimating and focusing mirrors, respectively.

In addition to astigmatism, coma is another aberration that must be considered. Coma causes broadening of spectral line profiles, which can significantly degrade resolution. To meet the requirements for coma correction, Shafer proposed specific coma-correction conditions for C-T type spectrometers [32]:

(10) $$\frac{\sin(\varphi_{2}/2)}{\sin(\varphi_{1}/2)}=\frac{{R_{2}}^{2}}{{R_{1}}^{2}}\frac{\cos(\varphi_{2}/2)\cos^{3}\alpha }{\cos(\varphi_{1}/2)\cos^{3}\beta }$$

Reflective grating satisfies the grating equation along the tangential plane and the law of reflection along the sagittal plane, which enables the determination of $L_{\mathrm{FD}}$, the distance between the focusing mirror and the detector, in both directions [33]:

(11) $$S_{S}=L_{\mathrm{FDS}}=\frac{R_{1}R_{2}L_{\mathrm{SC}}}{2L_{\mathrm{SC}}(R_{1}\cos\varphi_{2}+R_{2}\cos\varphi_{1})-R_{1}R_{2}}$$

(12) $$S_{T}=L_{\mathrm{FDT}}=\frac{R_{1}R_{2}L_{\mathrm{SC}}}{2L_{\mathrm{SC}}(R_{1}\sec\varphi_{2}+R_{2}\sec\varphi_{1}\frac{\cos^{2}\alpha }{\cos^{2}\beta })-R_{1}R_{2}\frac{\cos^{2}\alpha }{\cos^{2}\beta }}$$

By requiring equality between the tangential and sagittal image distances—thereby satisfying the zero-order astigmatism-free condition $S_{S}=S_{T}=L_{\mathrm{FD}}$, the distance $L_{\mathrm{SC}}$ between the slit and the collimating mirror can be calculated:

(13) $$L_{\mathrm{SC}}=\frac{R_{1}R_{2}(\frac{\cos^{2}\alpha }{\cos^{2}\beta }-1)}{2\left[R_{1}(\sec\varphi_{2}-\sec\varphi_{1})+R_{2}(\sec\varphi_{1}\frac{\cos^{2}\alpha }{\cos^{2}\beta }-\cos\varphi_{1})\right]}$$

The zero-order astigmatism-free condition only corrects aberrations at the central wavelength, whereas the first-order astigmatism-free condition is applied to determine the distance $L_{\mathrm{GF}}$ between the grating and focusing mirror for the edge wavelengths:

(14) $$L_{\mathrm{GF}}=R_{2}\cos\varphi_{2}\left(1-\frac{\frac{\partial S_{T}}{\partial \beta }}{\frac{\partial S_{S}}{\partial \varphi_{2}}-\frac{\partial S_{T}}{\partial \varphi_{2}}}\right)$$

(15) $$\frac{\partial S_{S}}{\partial \varphi_{2}}=\frac{2S_{S}L_{\mathrm{SC}}R_{1}\sin\varphi_{2}}{2L_{\mathrm{SC}}(R_{1}\cos\varphi_{2}+R_{2}\cos\varphi_{1})-R_{1}R_{2}}$$

(16) $$\frac{\partial S_{T}}{\partial \varphi_{2}}=\frac{-2S_{T}L_{\mathrm{SC}}R_{1}\sec\varphi_{2}\tan\varphi_{2}}{2L_{\mathrm{SC}}(R_{1}\sec\varphi_{2}+R_{2}\frac{\cos^{2}\alpha }{\cos^{2}\beta }\sec\varphi_{1})-R_{1}R_{2}\frac{\cos^{2}\alpha }{\cos^{2}\beta }}$$

(17) $$\frac{\partial S_{T}}{\partial \beta }=\frac{-2S_{T}R_{2}(2L_{\mathrm{SC}}\sec\varphi_{2}-R_{1})\cos^{2}\alpha \tan\beta \sec^{2}\beta }{2L_{\mathrm{SC}}(R_{1}\sec\varphi_{2}+R_{2}\frac{\cos^{2}\alpha }{\cos^{2}\beta }\sec\varphi_{1})-R_{1}R_{2}\frac{\cos^{2}\alpha }{\cos^{2}\beta }}$$

Dane R. Austin [34] suggested that the distance $L_{\mathrm{CG}}$ from the collimator to the grating has a relatively minor impact on imaging quality, typically equal to half of $R_{1}$.

To verify the feasibility of the C-T type spectrometer system, its parameters were calculated; the spectrometer is specified with a working wavelength range covering the dispersive objective lens, from 400 to 700 nm and a central wavelength of 550 nm. The grating has a line density of 600 lp/mm, and the system assigns an incident angle α of 8°. Based on the grating equation, a diffraction angle of 27.98° is generated. Based on these parameter settings, analytical constraints targeting different optical aberrations were successively introduced in the design process to clarify the physical basis of each parameter and its role in aberration control. First, system coma was constrained using the coma-correction condition of the C-T configuration proposed by Shafer, which was used to determine the off-axis angle and thereby achieve coma correction at the central wavelength. On this basis, the zero-order astigmatism-free condition was applied to correct astigmatism at the central wavelength, enabling the calculation of the geometric distances from the entrance slit to the collimating mirror and from the focusing mirror to the detector. Furthermore, the first-order astigmatism-free condition was introduced to compensate astigmatism at the edge wavelengths, thereby determining the relative position between the grating and the focusing mirror. Through this design procedure, effective suppression of coma, zero-order astigmatism, and first-order astigmatism was achieved sequentially within the specified working wavelength range. All calculations were performed in MATLAB (version R2023b), and the resulting optical structural parameters are summarized in Table 3.

## 3. Design of a Large-Angle Spectral Confocal Optical System

The large-angle spectral confocal optical system comprises two main subsystems: a dispersive objective lens and a spectrometer. Optical signals are routed and coupled between the subsystems using Y-type fiber optic couplers. The dispersive objective lens serves as a crucial element in spectral confocal sensors, determining both the measurement range and the angular measurement capability of the sensor. During the design process, a balance must be struck between enlarging the measurement range and increasing the NA. An excessively large measurement range reduces light energy utilization efficiency and imaging signal-to-noise ratio. Increasing the NA can improve light energy utilization efficiency and the signal-to-noise ratio to a certain extent, but it also complicates aberration correction. The spectrometer establishes a mapping between the focused wavelength and the corresponding position, and its performance directly governs the resolution of the spectral confocal sensor. This study adopts a C-T reflective configuration and employs a 14 μm × 200 μm line-scan detector. To achieve high spectral resolution, aberration correction is required to minimize the influence of astigmatism on light energy utilization efficiency and system resolution.

### 3.1. Design of the Dispersive Objective Lens

During the design process, evaluation of the dispersive objective lens emphasizes spot size and beam focusing, while image sharpness, distortion, and astigmatism are disregarded; consequently, spherical aberration is identified as the primary aberration requiring correction. On-axis monochromatic spherical aberration enlarges the spot size at the image plane, broadens the spectral profile, and degrades the axial resolution of the sensor, thus requiring precise correction. To mitigate this effect, the lens can first be divided into multiple elements. By evenly distributing the optical power among four to five elements, the spherical aberration can be reduced to approximately 10–15% of that of a single lens, while maintaining the original NA and total optical power of the system [35]. Furthermore, the separated elements can be combined into a cemented doublet to further suppress spherical aberration.

Zemax optical design software was used to perform simulation and optimization of the dispersive objective lenses. Since the dispersive objective lens in a spectral confocal system must intentionally preserve and precisely control axial chromatic aberration to establish a one-to-one correspondence between wavelength and axial positioning, conventional imaging performance metrics, such as modulation transfer function (MTF) and wavefront aberration, are not suitable as primary optimization criteria. Therefore, the root-mean-square (RMS) spot radius was selected as the main merit function to evaluate monochromatic aberration control at each working wavelength.

**Figure 4:**
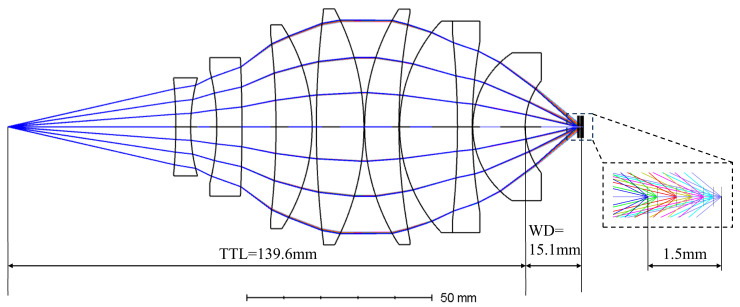
Schematic of the optimized dispersive objective lens structure. In the lower-right enlarged region, rays of different colors from left to right correspond to different wavelengths: blue for 430 nm, green for 450 nm, red for 500 nm, yellow for 550 nm, pink for 600 nm, cyan for 650 nm, and violet for 700 nm.

A multi-configuration optimization was performed over the visible wavelength range of 430–700 nm, with seven reference wavelengths selected. Except for the second wavelength, the reference wavelengths were chosen at 50 nm intervals, and the principal wavelength was set at 550 nm. The object-side numerical aperture was fixed at 0.22. During the optimization, the axial distances from the last surface of the dispersive objective lens to the best focal planes for each reference wavelength were treated as multi-configuration design variables, in order to maintain a linear relationship between wavelength and focal shift. At the same time, the system numerical aperture was constrained to limit the maximum allowable object angle. Lens curvatures, thicknesses, and inter-lens air gaps were used as optimization variables. The optimization performance was evaluated by the RMS spot radius at the best focal plane corresponding to each reference wavelength, so that the RMS spot radius at each focal plane remained below the corresponding Airy disk radius. After the preliminary optimization, a Hammer optimization was applied to refine the structural material parameters. In combination with the distributions of refractive indices and Abbe numbers, certain lens materials were replaced or reconfigured to improve aberration balance and chromatic compensation under multi-wavelength conditions. The optimized dispersive objective lens structure is shown in Figure 4, consisting of six singlet lenses and one cemented doublet, all with spherical surfaces. The image-side numerical aperture of the system is 0.645, the working distance exceeds 15 mm, the axial dispersion range reaches 1.5 mm, and the total system length is 139.6 mm.

**Figure 5:**
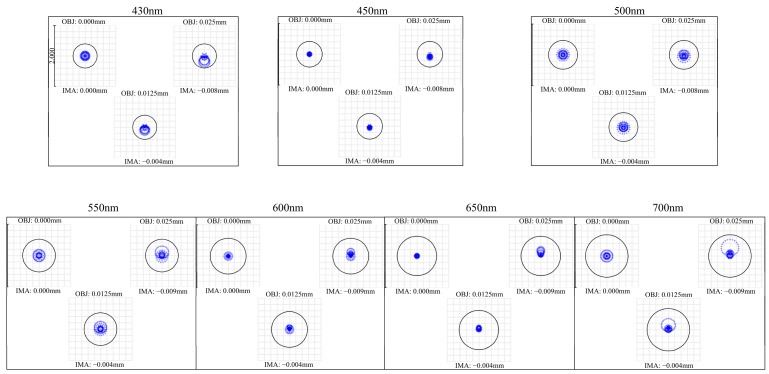
Spot diagrams at different wavelengths. Round circles indicate corresponding Airy disks at each wavelength.

**Figure 6:**
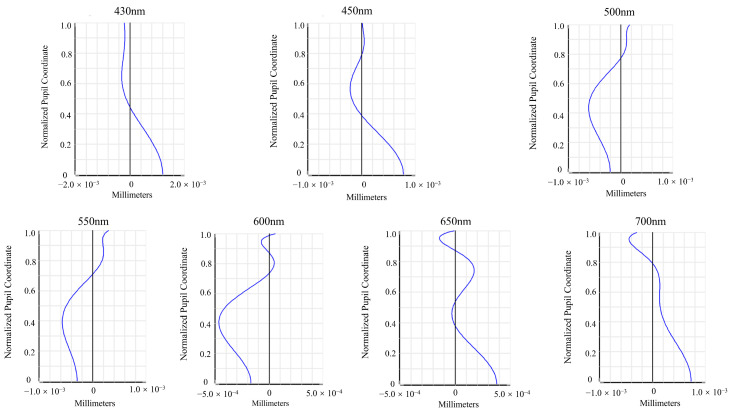
Spherical aberration curves at different wavelengths.

The spot diagrams and spherical aberration curves at wavelengths of 430, 450, 500, 550, 600, 650, and 700 nm are shown in Figure 5 and Figure 6, respectively. At all wavelengths, the RMS spot radius at the focal plane remains below the corresponding Airy disk radius. The peak spherical aberrations at these wavelengths are 1.207, 0.780, −0.618, −0.570, −0.468, 0.378, and 0.732 μm, respectively, indicating well-controlled spherical aberration and excellent imaging performance across the visible spectrum.

**Figure 7:**
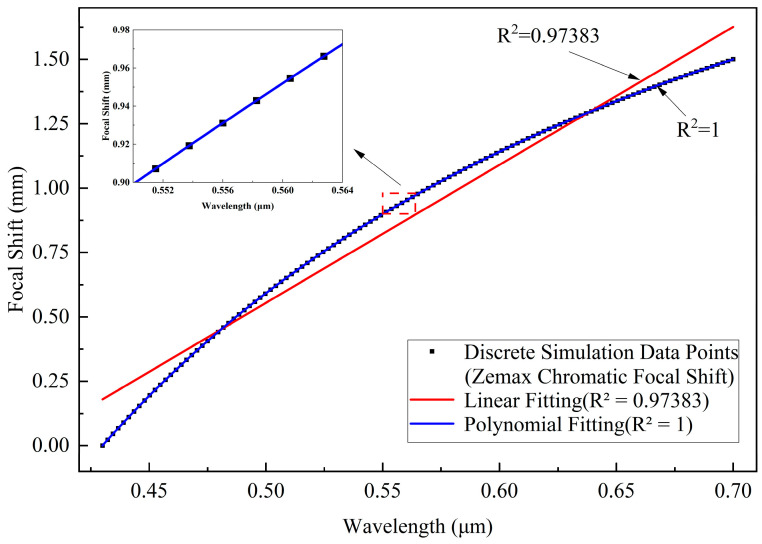
Wavelength versus axial dispersion fitting curve.

The spectral confocal sensor ultimately yields a unique relation between the measured position and the peak wavelength. For the dispersive objective lens, improved proportionality between axial dispersion and wavelength contributes to higher measurement accuracy. The proportional relationship of axial dispersion with wavelength was obtained via least-squares fitting, as shown in Figure 7. The black dots represent discrete chromatic focal shift (CFS) data exported from Zemax, which directly characterize the axial dispersion range of the dispersive objective lens. As can be observed from the CFS distribution, the total axial dispersion exceeds 1.5 mm over the 430–700 nm wavelength range, satisfying the design requirement of the spectral confocal system. These discrete CFS data serve as the raw and initial dataset for both linear and polynomial fittings. The red solid line denotes the linear fit to these data points, which exhibits an axial dispersion of 1.5 mm over the 430–700 nm wavelength range ($R^{2}$ = 0.97383). Deviations from this linear fit are most pronounced at the short- and long-wavelength ends and near the central wavelength, primarily due to the dispersion characteristics of the glass materials. At the spectrum edges, the refractive index varies more significantly with wavelength, making it challenging to maintain a highly linear axial dispersion across the full wavelength range. The blue curve in Figure 7 represents the polynomial fit. To obtain this curve, a fifth-order polynomial fitting method is employed, which passes exactly through all black data points, yielding a coefficient of determination $R^{2}$ = 1. Specifically, the resulting relationship can be expressed in the form of the following fifth-order polynomial equation:

(18) $$LD=-52.704\lambda^{4}+146.771\lambda^{3}-159.341\lambda^{2}+82.569\lambda -15.910$$

where LD represents the position of the relative focal point, and λ represents the wavelength. The polynomial curve effectively characterizes the mapping between wavelengths and their respective focal positions. However, variations in slope across the spectrum result in minor changes to the resolution performance of the dispersive objective lens.

In this context, the axial dispersion nonlinearity ($R^{2}$ = 0.97383) indicates that the sensitivity of axial position to wavelength is not uniform across the entire spectral range. As a result, the same wavelength measurement error can produce different magnitudes of axial position error at different wavelengths, leading to a non-uniform axial resolution across the measurement range, particularly near the spectrum edges. In practice, this effect can be mitigated through experimental calibration to establish the true wavelength–axial position mapping, combined with signal processing techniques such as spectral filtering and peak wavelength estimation; with appropriate calibration and post-processing, the axial dispersion nonlinearity does not constitute a major limitation for depth measurement accuracy.

Since the spectral information received by the detector is reflected from the measured object, passes through the dispersive objective lens a second time, and is finally delivered to the spectrometer via the Y-type fiber optic coupler. Therefore, it is necessary to analyze the propagation of light through the dispersive objective lens a second time to determine whether all rays carrying information from the measured object can enter the optical fiber. A mirror is placed at the focal plane corresponding to a specific wavelength to simulate the target object’s surface. The beam traverses the dispersive objective lens, reflects off the mirror, and returns through the dispersive objective lens to form an image at the position of the light source.

**Figure 8:**
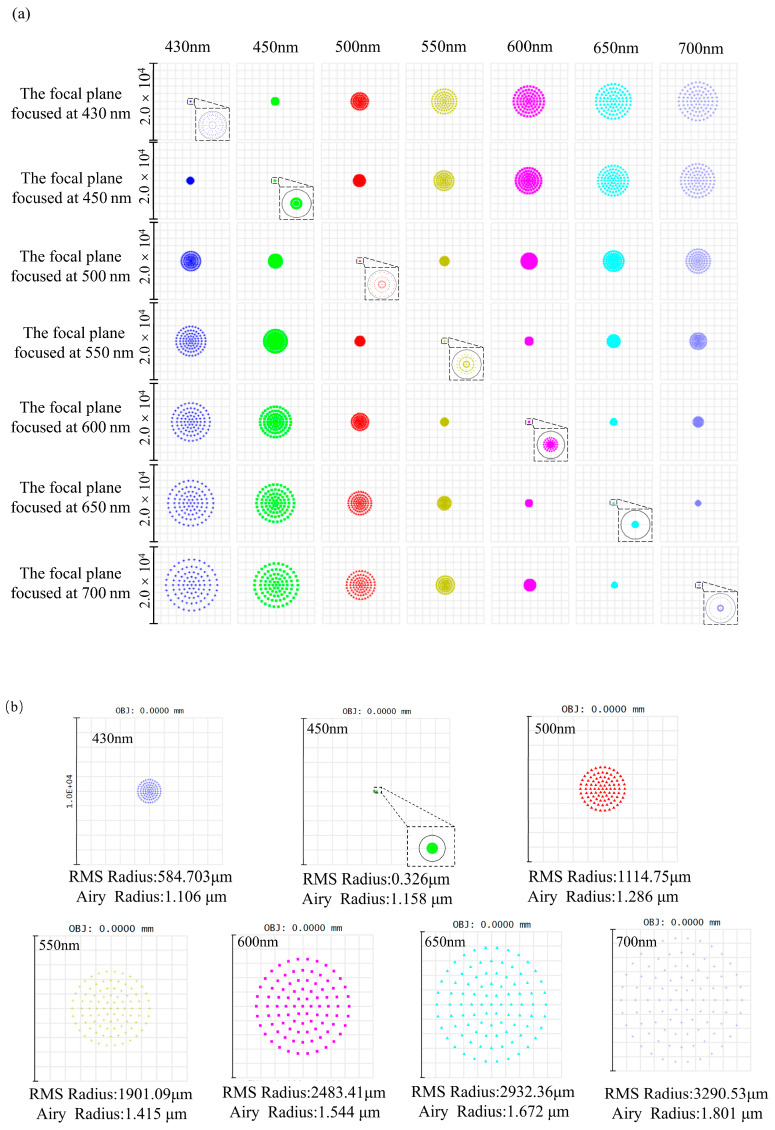
Spot diagram distribution at the focal planes corresponding to different reference wavelengths. The round circles indicate the corresponding Airy disks. (**a**) Matrix of spot diagrams showing the distribution of all working wavelengths at each wavelength-specific focal plane. (**b**) Representative spot diagram when the system is focused at 450 nm.

Figure 8 illustrates the spot distributions on the focal planes. (a) shows a matrix of spot diagrams of all working wavelengths evaluated at the focal plane corresponding to each reference wavelength. It can be observed that when the reflector is positioned at the optimal focal plane of a given reference wavelength, only that wavelength exhibits the minimum spot size, which is smaller than the corresponding Airy disk radius. All other wavelengths are defocused to varying degrees, and their spot sizes increase progressively as the wavelength deviates further from the focal wavelength. (b) presents a representative case in which the reflector is positioned at the focal plane corresponding to 450 nm. Under this condition, the RMS spot radius at 450 nm is 0.326 μm, which is smaller than the corresponding Airy disk radius of 1.158 μm, indicating diffraction-limited focusing at the design wavelength. At wavelengths of 430, 500, 550, 600, 650, and 700 nm, the spot diagrams are defocused, showing large and diffuse spots. At the wavelength of maximum deviation (700 nm), the RMS spot radius increases to 3290.53 μm. Since the optical fiber has a diameter of 50 μm, light at the focal wavelength and nearby wavelengths can all enter the fiber. As the wavelength deviates further from the focal wavelength, the optical energy coupled into the fiber decreases sharply. Therefore, the designed dispersive objective lens exhibits pronounced axial dispersion characteristics, enabling the spatial separation of different wavelengths.

An ideal plane mirror corresponds to strict normal incidence and coaxial retroreflection. However, in practical droplet measurements, when the local surface tilt is within the allowable object angle set by the image-side NA of the dispersive objective lens, a portion of the reflected light can still be collected and coupled back into the fiber. In this case, spectral confocal sensor primarily relies on the spectral peak wavelength of the reflected light rather than its intensity. Therefore, surface tilt mainly affects the signal amplitude, with relatively limited impact on the accuracy of axial position demodulation. By combining multi-point surface sampling with global surface fitting, the influence of local signal attenuation on the reconstructed droplet profile and curvature inversion can be effectively suppressed. The plane mirror model and measurement strategy adopted in this work primarily apply to droplet surface regions within the image-side NA allowable range, where their validity is ensured by both the system design and the spectral confocal sensor mechanism. When the droplet surface tilt further increases beyond the maximum allowable object angle allowed by the system’s image-side NA, the reflected light can no longer be efficiently returned to the sensor. For such situations, previous studies have shown that introducing a collimating lens in the return path along with a retroreflector can redirect the reflected light back into the sensor, enabling oblique-incidence spectral confocal measurements. This method can be regarded as a compensation and extension strategy for angles exceeding the image-side NA allowable range [36].

### 3.2. Design of the Spectrometer

The spectrometer primarily comprises the collimating mirror, the diffraction grating, and the focusing mirror. During design, tangential aberrations are primarily considered to achieve high spectral resolution, while the sagittal direction is sometimes assigned zero weight to further optimize image quality along the tangential plane. In off-axis reflective systems, astigmatism is a primary aberration. Although astigmatism does not directly affect spectral resolution, in C-T type spectrometers, the miniaturization of the device partially limits the optical energy throughput of the spectrometer, and spectral line broadening caused by astigmatism further increases energy loss. Table 3 lists the fundamental parameters of the C-T type spectrometer under astigmatism-free conditions.

**Figure 9:**
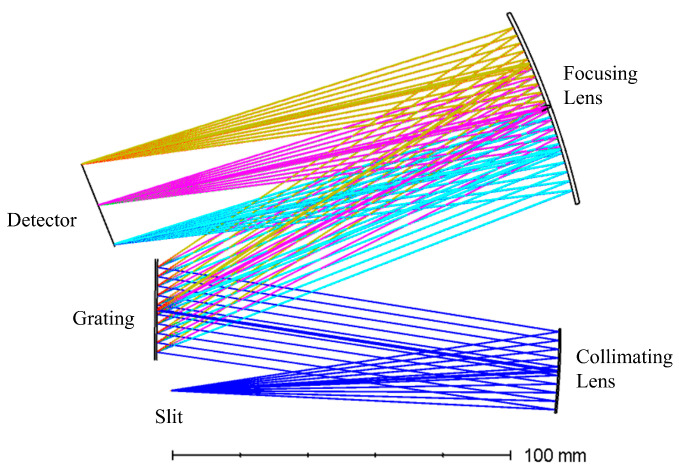
Optimized the optical path of the C-T type spectrometer. Rays of different colors represent light of different wavelengths. From above to below at the detector: yellow for 700.54 nm, red for 700 nm, pink for 550.54 nm, green for 550 nm, cyan for 400.54 nm, and blue for 400 nm.

The optical path of the C-T type spectrometer was optimized using Zemax. Variables included the distance from the focusing mirror to the image plane, the tilt angle of the image plane, the tilt angle and decenter of the collimating mirror, the tilt angle of the grating, and the tilt angle and decenter of the focusing mirror. The RMS spot radius along the slit-width direction (Y-direction) was optimized. The default merit function was set to the Y-direction RMS spot radius, with a weight ratio of 1:0 between the tangential and sagittal directions. The optimized spectrometer optical path is shown in Figure 9.

**Figure 10:**
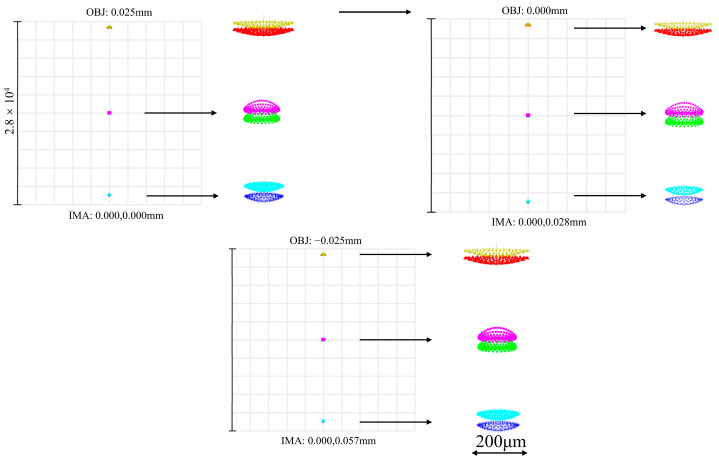
Spot diagram of the C-T type spectrometer under astigmatism-free conditions. Rays of different colors represent light of different wavelengths. From top to bottom: yellow for 700.54 nm, red for 700 nm, pink for 550.54 nm, green for 550 nm, cyan for 400.54 nm, and blue for 400 nm.

The C-T type spectrometer uses the spot diagram as the evaluation criterion. The optical fiber collects light reflected from the measured surface and delivers it to the detector, with the object height set according to the fiber core diameter of 50 µm. Figure 10 shows the spot diagram of the spectrometer under astigmatism-free conditions. According to the Rayleigh criterion, two points are considered resolvable when the center of one point’s diffraction pattern coincides with the first dark ring of the other. In general, spots in the spot diagram are considered resolvable when their overlap does not exceed one-half. Analysis of the spot diagram reveals noticeable overlap at the central wavelength; however, at a wavelength of 550 nm, the overlap along the Y-direction is less than 30%. At a wavelength of 700 nm, the overall spectral broadening slightly surpasses 200 μm, but the core region remains within this range. Consequently, the system’s broadening in the X-direction is effectively maintained within the single pixel (200 μm). These results demonstrate that the spectrometer, under astigmatism-free conditions, can resolve two wavelengths separated by 0.54 nm, thereby satisfying the design resolution requirement. Furthermore, astigmatism is effectively controlled across the entire wavelength range.

**Figure 11:**
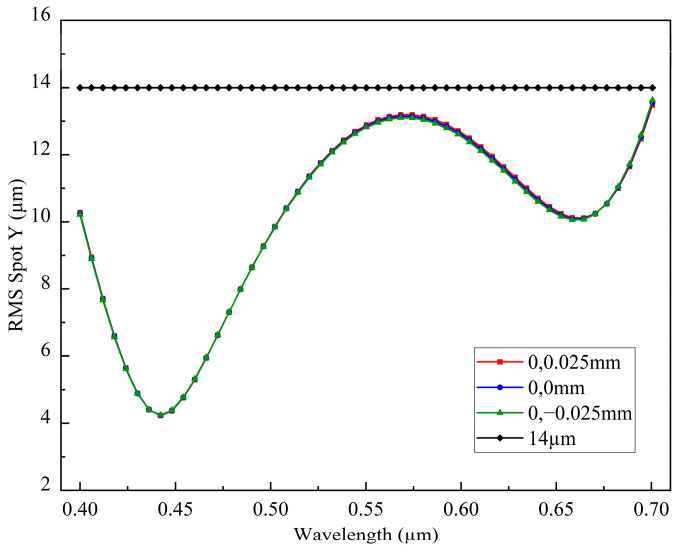
Relationship between Y-direction RMS spot radius and wavelength.

Figure 11 shows the variation in the Y-direction RMS spot radius across the wavelength range. It can be observed that across the 400–700 nm spectral range. The Y-direction RMS spot radius consistently remains below the single-pixel size of 14 µm, demonstrating the excellent imaging performance of the spectrometer.

**Figure 12:**
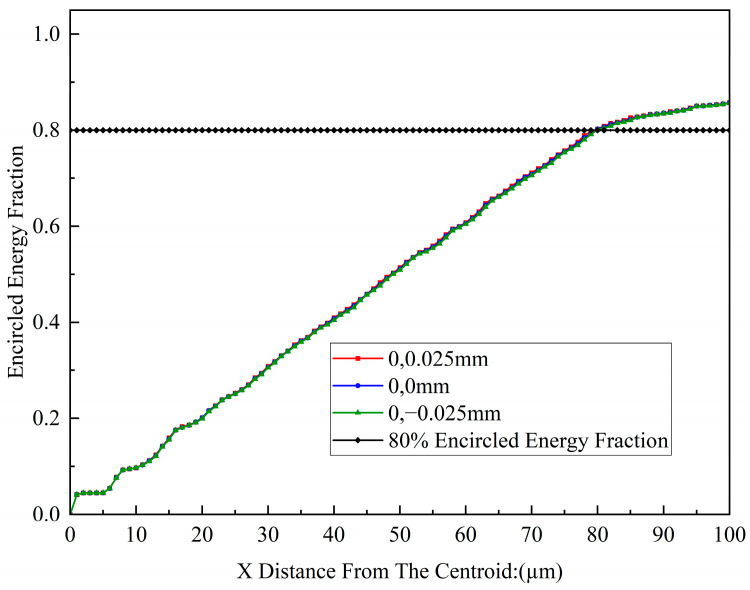
Encircled energy along the X-direction on the image plane of the spectrometer.

The system requires the spectrometer detector to capture the maximum possible light intensity while maintaining spectral resolution; therefore, it is essential to control the beam dimensions in the non-dispersive direction. The analysis of the encircled energy for an X-direction radius of 100 µm is presented, and the average encircled energy distribution is shown in Figure 12.

In summary, the designed C-T type spectrometer, under astigmatism-free conditions, achieves high spectral resolution and excellent imaging performance across the working wavelength range. The system effectively suppresses astigmatism-induced energy loss while maintaining spectral resolution, with an average encircled energy exceeding 80%, thereby meeting the performance requirements of compact spectrometers.

## 4. Tolerance Analysis

Tolerance analysis is a critical process in assessing the manufacturability of an optical system after the completion of its design. It serves to assess the system’s feasibility during actual machining and assembly, ensuring that the optical performance remains within design specifications under manufacturing and alignment errors. In order to guarantee that the dispersive objective lens and the spectrometer maintain high imaging quality and manufacturability after machining and assembly, this study performs a tolerance analysis on the optimized dispersive objective lens and spectrometer.

The dispersive objective lens is assessed using spot diagrams at different wavelengths, with the requirement that the RMS spot radius following tolerance allocation does not exceed a single detector pixel (14 µm). The corresponding tolerance allocation for the dispersive objective lens is summarized in Table 4. It can be seen that, for the dispersive objective lens system, surface thickness, surface tilt, and material refractive index tolerances have a significant impact on overall system performance. The surface tolerances of the subsequent four lenses within the dispersive objective lens are also influential. Based on the tolerance sensitivity analysis, these critical parameters were therefore tightened: the surface thickness tolerance to 0.0125 mm, the surface tilt tolerance to within ±0.5′, and the material refractive index tolerance to ±0.0005. This allocation balances performance stability with practical manufacturability.

Employing the Monte Carlo analysis with spot diagrams at each wavelength as the evaluation criterion, the results are summarized in Table 5. After tolerance allocation, the dispersive objective lens exhibits a 90% probability that the RMS spot radius at each wavelength remains smaller than a single detector pixel (14 µm). For comparison, the spot diameter of existing large-angle spectral confocal dispersive objective lenses typically ranges from 5 to 15 µm. The designed spot diameter is smaller than this range, and the tolerance analysis results meet the design specifications.

The RMS spot radius Y-direction spot radius is employed as the evaluation metric in the spectrometer’s tolerance analysis. By defining and allocating the surface machining and assembly tolerance of key optical components, the analysis evaluates system performance under the influence of manufacturing and alignment errors. Table 6 shows the tolerance allocation of the spectrometer. For the spectrometer, tolerance analysis indicates that the surface thickness is the dominant factor influencing overall system performance. Variations in this parameter were found to have a pronounced effect on optical alignment and spectral imaging quality. Accordingly, based on the tolerance sensitivity results, the surface thickness tolerance was tightened to 0.0125 mm in order to ensure stable system performance and reliable spectral measurement, while remaining compatible with practical manufacturing and assembly capabilities.

Monte Carlo analysis was conducted on the spectrometer. Under the initial design conditions, the RMS spot radius in the Y-direction was 12.85781 µm. Statistical analysis was performed with 200 samples, and the tolerance analysis results for the spectrometer are presented in Table 7. Results show that, under the astigmatism-free condition, 90% of samples have an RMS spot radius in the Y-direction within 13.23686 µm. This value is smaller than a single detector pixel (14 µm). These results demonstrate that the spectrometer maintains high imaging quality even when tolerance effects are considered, thereby satisfying the design resolution requirements.

After completing the optical system design and performing the tolerance analysis, its performance was compared with that of conventional commercial spectral confocal optical systems, as summarized in Table 8 (e.g., CL1-MG210, CL3-MG70, IF2405-1) [37,38]. Although these systems achieve nanometer-scale axial resolution, their measurement range and maximum allowable object angles are relatively limited. In contrast, the spectral confocal system designed in this study achieves a relatively large measurement range (1.5 mm) and large-angle measurement capability (±40°), with an axial resolution at the micrometer level (3 µm). While the resolution is lower than that of traditional nanometer-scale systems, considering the dimensional scale and surface variation characteristics of droplets, micrometer-scale axial resolution is sufficient for droplet profile measurements.

## 5. Conclusions

Spectral confocal sensors are a high-precision, non-contact method for profile measurement. To enable large-angle measurements of droplet surfaces, this study presents an optimized optical system design for spectral confocal sensors. The dispersive objective lens, combining positive and negative lenses, provides 1.5 mm of axial dispersion over 430–700 nm, with a maximum allowable object angle of ±40°. RMS spot radii at the focal plane remain below the diffraction limit, and the wavelength-to-axial position relationship exhibits $R^{2}$ = 0.97383. A C-T type spectrometer was employed, with astigmatism-free conditions analyzed and optical component parameters determined. The optimized spectrometer achieves 0.54 nm spectral resolution without additional optical elements, while RMS spot radii remain below the 14 µm detector pixel size. Tolerance analysis of the dispersive lens and spectrometer confirms good manufacturability during machining and assembly. Beyond droplet surface profile measurement, owing to its spectral confocal axial encoding mechanism and maximum allowable object angle, the proposed large-angle spectral confocal design can, in principle, be applied to surface profile measurements of complex geometries with large local surface orientations, including microchannels, micro-structured surfaces, and optical lens profiles. The present work mainly focuses on optical system design and simulation analysis, with the droplet surface tension inversion model based on static equilibrium assumptions. Therefore, the current method is not applicable to time-varying droplets. In future work, system construction and experimental validation will quantitatively evaluate performance, while also exploring extension to time-varying droplets to further verify feasibility and promote practical applications.

## Figures and Tables

**Table 1:** Design parameters of the spectral confocal sensor optical system.

Parameter	Value
Working wavelength	430–700 nm
Measurement range	≥1.5 mm
System resolution	≤3 μm
Maximum allowable object angle	≥±40°

**Table 2:** Design specifications of the dispersive objective lens.

Parameter	Value
Working wavelength	430–700 nm
Object-side NA	0.22
Image-side NA	≥0.645
Measurement range	≥1.5 mm
Working distance	>15 mm
Total system length	≤150 mm

**Table 3:** Basic parameters of the C-T type spectrometer.

Parameter	Value
Wavelength range	400–700 nm
Center wavelength	550 nm
Grating line density	600 lp/mm
Focal length of the collimating mirror	125 mm
Focal length of the focusing mirror	125 mm
Diameter of the entrance slit	50 μm
Pixel size	14 × 200 μm
Distance from the slit to the collimating mirror $L_{\mathrm{SC}}$	110.37 mm
Distance from the collimating mirror to the grating $L_{\mathrm{CG}}$	125.00 mm
Distance from the grating to the focusing mirror $L_{\mathrm{GF}}$	105.64 mm
Distance from the focusing mirror to the image plane $L_{\mathrm{FD}}$	146.86 mm

**Table 4:** Tolerance allocation parameters of the dispersive objective lens.

Tolerance Type	Tolerance Items	Surface	Tolerance Value	Remarks
Surface tolerance	Radius of curvature	All surfaces	±2 fringe	/
	Thickness	1–2, 3–4, 5–6, 6–7, 8–10, 11–12, 13–14, 14–15	±0.0125 mm	Other surfaces: 0.0375 mm
	Surface tilt	4, 5, 7, 8, 9, 10, 12, 13	±0.5′	Other surfaces: ±1′
	Surface decenter	9	±0.005 mm	Other surfaces: ±0.01 mm
Material tolerance	Refractive index	5, 7, 9, 11, 12	±0.0005	Other surfaces: ±0.001
	Abbe number	/	±0.1%	/
Assembly tolerance	Component decenter	/	/	Other surfaces: ±0.01 mm
	Component tilt	3–4, 5–6	±0.05′	Other surfaces: ±1′

**Table 5:** Monte Carlo analysis results of the dispersive objective lens.

Wavelength	90% Probability in Monte Carlo Analysis
430 nm	2.99431 µm
450 nm	2.90495 µm
500 nm	2.85618 µm
550 nm	2.57953 µm
600 nm	2.53159 µm
650 nm	2.48378 µm
700 nm	2.36030 µm

**Table 6:** Tolerance allocation parameters of the spectrometer.

Tolerance Type	Tolerance Item	Surface	Tolerance Value	Remarks
Surface tolerance	Radius of curvature	All surfaces	±1 fringe	/
	Thickness	1–2 (+), 5–6 (+), 6–7 (−), 9–10 (−)	±0.0125 mm	Other surfaces: ±0.0375 mm
Assembly tolerance	Component decenter	10	±0.005 mm	Other surfaces: ±0.01 mm
	Component tilt	2, 6 (−), 10 (+)	±0.05′	Other surfaces: ±1′

**Table 7:** Monte Carlo analysis results of the spectrometer.

Probability	90% Probability RMS Spot Radius in the Y-Direction in Monte Carlo Analysis
90%	13.23686 µm
80%	12.85216 µm
50%	11.95182 µm
20%	11.15943 µm
10%	10.58506 µm

**Table 8:** Comparison of commercial spectral confocal systems and the proposed large-angle system.

Parameter	Measuring Range	Working Distance	Maximum Allowable Object Angle	Axial Resolution	Spot Size
CL1-MG210	0.15 mm	3.3 mm	±42°	1.33 nm	2.7 µm
CL3-MG70	1.4 mm	12.2 mm	±25°	10 nm	11.9 µm
IF2405-1	1 mm	10 mm	±30°	8 nm	8 µm
This Article	1.5 mm	15.1 mm	±40°	3 µm	2.6 µm

## Data Availability

No new data were created.
